# Recurrent competition explains temporal effects of attention in MSTd

**DOI:** 10.3389/fncom.2012.00080

**Published:** 2012-10-05

**Authors:** Oliver W. Layton, N. Andrew Browning

**Affiliations:** Center for Computational Neuroscience and Neural Technology, Boston UniversityBoston, MA, USA

**Keywords:** attention, heading, model, motion processing, MST, navigation, optic flow

## Abstract

Navigation in a static environment along straight paths without eye movements produces radial optic flow fields. A singularity called the focus of expansion (FoE) specifies the direction of travel (heading) of the observer. Cells in primate dorsal medial superior temporal area (MSTd) respond to radial fields and are therefore thought to be heading-sensitive. Humans frequently shift their focus of attention while navigating, for example, depending on the favorable or threatening context of approaching independently moving objects. Recent neurophysiological studies show that the spatial tuning curves of primate MSTd neurons change based on the difference in visual angle between an attentional prime and the FoE. Moreover, the peak mean population activity in MSTd retreats linearly in time as the distance between the attentional prime and FoE increases. We present a dynamical neural circuit model that demonstrates the same linear temporal peak shift observed electrophysiologically. The model qualitatively matches the neuron tuning curves and population activation profiles. After model MT dynamically pools short-range motion, model MSTd incorporates recurrent competition between units tuned to different radial optic flow templates, and integrates attentional signals from model area frontal eye fields (FEF). In the model, population activity peaks occur when the recurrent competition is most active and uncertainty is greatest about the relative position of the FoE. The nature of attention, multiplicative or non-multiplicative, is largely irrelevant, so long as attention has a Gaussian-like profile. Using an appropriately tuned sigmoidal signal function to modulate recurrent feedback affords qualitative fits of deflections in the population activity that otherwise appear to be low-frequency noise. We predict that these deflections mark changes in the balance of attention between the priming and FoE locations.

## Introduction

Neurons in the dorsal medial superior temporal area (MSTd) of primate visual cortex selectively respond to radially expanding random dot patterns that span large parts of the visual field (Duffy and Wurtz, 1991, 1995). Gibson (1979) noted that animals experience radially expanding patterns of motion during navigation along a straight path, in the absence of eye movements. The center of the radial motion is known as the *focus of expansion* (FoE) and defines the direction of travel (heading) during locomotion. Primate MSTd neurons exhibit selectivity to the FoE location, and researchers have proposed that these cells are important for heading perception during visually guided navigation (Duffy and Wurtz, 1991; Born and Bradley, 2005). Neurophysiological data indicate that primate heading sensitive MSTd cells receive feedforward projections from cells sensitive to local motion in V1 via cells that integrate motion over a large receptive field in primate medial temporal area, MT (Eifuku and Wurtz, 1998; Berezovskii and Born, 2000; Orban, 2008). Objects that move independently of the observer in the environment induce distinct patterns of motion that differ from the patterns experienced by the observer in the object's absence. The object induces its own FoE when the independently moving object approaches the observer. Depending on whether the object approaches or recedes, the object size, and other contextual information, the observer may shift his focus to different aspects of the environment (Kishore et al., 2011; Wann et al., 2011). While the effects of visual attention have been studied in ventral areas (e.g., V4) and early dorsal areas (e.g., MT), the role attention plays on MSTd neurons and visually guided navigation has only been recently examined.

### Attention affects the gain of individual neurons

Visual attention has been characterized in the psychological literature as a spatial “spotlight” with limited resources (Posner et al., 1980), which enhances a subject's visual search and luminance detection performance (Yeshurun and Carrasco, 1998) and may reduce the subject's response latency (Treisman, 1998). Recent neurophysiological experiments have demonstrated that attention directly modulates the activity of individual neurons throughout the occipital and parietal cortices (Corbetta and Shulman, 2002). In primate visual area V4, the response of neurons increases to both an attended “target” object and proximally located behaviorally irrelevant objects (Connor et al., 1997). When low contrast objects are presented within the receptive field, attention increases the activity of neurons to the same levels that would occur in response to objects of higher contrast (Reynolds and Chelazzi, 2004). Neural signals, which modulate the gain of visual neurons in extrastriate cortex, may originate from top–down sources further up the visual pathways, since changes in a neuron's activity take ~70 ms (Martinez et al., 1999) after the visual display is presented to the subject. The apparent response gain modulation observed in visual neurons is consistent with the idea of resource limitation or normalization of activity (Reynolds and Heeger, 2009), since the increased neural activity to an attended object may accompany a diminished response to competing objects (Reynolds and Desimone, 2003).

### Multiplicative or non-multiplicative attention

While attentional gain modulation has been well documented, exactly how an attentional signal acts on baseline neural responses remains unclear. Some researchers have proposed that spatial attention *multiplicatively* influences sensory bottom–up signals such that the size and position of the receptive field of a neuron do not change, but the preferred response and tuning curve distributions do (Martinez et al., 1999; Treue and Martnez Trujillo, 1999; McAdams and Maunsell, 2000; Williford and Maunsell, 2006). For instance, the response to the preferred motion direction of single neurons in primate visual area MT has been shown to increase when the monkey attends similar directions of motion outside the cell receptive field, and the response to anti-preferred motion decreases—suggestive of multiplicative changes in the tuning curves (Martinez-Trujillo and Treue, 2004). Others have proposed that attention acts *non-multiplicatively* on the sensory signal, which may change not only the tuning properties of neurons but also the spatial extent of their responses (Womelsdorf et al., 2006, 2008). For instance, the receptive fields of MT neurons have been shown to shift depending on whether the subject attends objects inside or outside the receptive field.

### MSTd and attention

**Figure 1:**
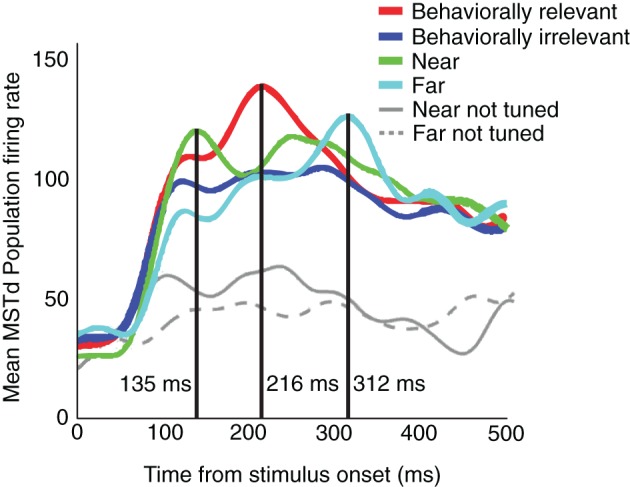
**Mean MSTd population firing rates,** in the behaviorally relevant (red), behaviorally irrelevant (blue), near (green), and far (cyan) conditions. The timing of the mean MSTd population responses in the near (135 ms), behaviorally relevant (216 ms), and far (312 ms) experimental conditions, respectively, after the optic flow display appeared was approximately linear. Data extracted from Figures 2C,D of Dubin and Duffy (2009) and combined into a single figure.

Dubin and Duffy (2007, 2009) investigated the responses of single neurons in MSTd when monkeys were presented with radial motion patterns and were primed to attend locations of the visual field some distance from the FoE. The researchers found that neurons selective for the FoE position showed an increased response when the monkeys fixated the center of the visual display and had to later saccade to the FoE location (*behaviorally relevant condition*). Covert attention was assumed to travel between the fixation and FoE locations during each trial because the FoE appeared randomly in one of eight locations at 30° eccentricity about the fixation point. In *behaviorally irrelevant* trials, an attentional prime appeared and disappeared in one of the FoE locations prior to the optic flow display onset and the monkeys had to saccade to the prime location after the optic flow presentation. The prime did not always coincide with the forthcoming optic flow FoE location. The firing rate of neurons tuned to the FoE position was enhanced, when the prime was close to the FoE compared to when it was far away. Figure 1 shows the MSTd population firing rate results for the behaviorally relevant (red) and irrelevant conditions (blue), averaged across all neurons, showing an effect for behaviorally relevant or irrelevant trials. This plot is derived from Figures 2C,D in Dubin and Duffy (2009). Figure 1 also shows the effects of distance between the attentional prime and the FoE in the behaviorally irrelevant condition, where the prime was located at 0° (near, green) or 60° (far, cyan) eccentricity. The timing of the peak average population response of MSTd neurons was related, almost linearly, to the distance in visual angle between the prime and the FoE (*r*^2^ = 0.89). The green, red, and cyan curves peak at 135, 216, and 312 ms, respectively. These peaks correspond to the focus of “attention” at 0°, 30°, and 60° eccentricity relative to the monkey fixation point. Although the mean MSTd population firing rates exhibit distinct peaks when the prime appears near or far from the FoE location, behaviorally irrelevant trials collectively yielded a flatter population response (blue). Neurons that were not strongly tuned to the FoE location in the near (gray, solid) and far (gray, dashed) prime conditions showed lower mean population activities compared to neurons tuned to the FoE. Why does the mean MSTd population peak recede in time, and by what mechanisms?

This article introduces a computational model of MSTd to mechanistically explain why the mean MSTd population response peak recedes in time as the visuotopic distance traveled by attention increases.

Our neural model addresses the effects of spatial attention on the neural dynamics, peak population response latencies, and tuning curves of MSTd cells reported by Dubin and Duffy (2007, 2009). The model contains stages that correspond to primate visual areas V1, MT, MSTd, and FEF. In model V1, units detect local motion (Livingstone and Conway, 2003). To focus on the dynamics of MSTd, we employ an analytic motion vector representation in model V1. Model V1 projects to model MT, wherein we perform a long-range motion pooling over the model V1 responses over time. Cells in primate MT elicit aperture-resolved responses to large fields containing uniform velocity patterns. Model MT projects to model MSTd, which has units that respond to large patterns of visual motion (Duffy and Wurtz, 1991). We include model area FEF, which projects to MSTd, to provide top–down attentional signals.

The spatial priming paradigm of Dubin and Duffy involves saccadic planning, decision making, and expectation formation in monkey subjects. Area FEF in both non-human primates (Krauzlis, 2005) and humans (Corbetta et al., 1998; Corbetta and Shulman, 2002) has been strongly implicated in goal-oriented top–down selection and spatial orientation (Schall, 2004) in a tightly integrated ocular-motor and attention neural circuit that projects to MST (Colby et al., 1988). Our model predicts that the attentional signals that modulate MSTd neurons in the experiments of Dubin and Duffy originate in FEF. The effects of attention have been documented in earlier visual areas, such as V1 (Paradiso, 2002), however, there are no data from V1 (or MT) during the modeled experiments and so we do not attempt to address those attentional effects. We demonstrate that recurrent competition between units in MSTd explains the data of Dubin and Duffy (2007, 2009), irrespective of the particular attentional signal used. Deflections in the population temporal activity are predicted to signify dynamic shifts in neural activity between units sensitive to the FoE and the prime locations, in other words as attention shifts from the prime to the FoE.

## Materials and methods

Our simulation conditions mimic the monkey behavioral paradigm of Dubin and Duffy (2007) and Dubin and Duffy (2009). In their experiments, two rhesus monkeys viewed radial dot optic flow displays composed of 1000 dots moving at ~40°/s on a 90° tangent screen. Single cells were recorded from area MSTd 50–300 ms following the onset of the optic flow display. The optic flow FoE appeared in one of eight locations regularly spaced by 45° at 30° eccentricity around a centrally located fixation point. The monkeys maintained fixation for 2 s during a trial, then the optic flow display appeared for 1 s. Monkeys were required to fixate within a 2° × 2° window of the center of the screen and were rewarded for completing a saccade task. On *behaviorally relevant* trials, a grid of eight targets, each corresponding to a possible FoE location, followed the optic flow display and the monkeys had 500 ms to saccade to the target that marked the position of the FoE during the trial. Dubin and Duffy interleaved behaviorally relevant trials with those that were *behaviorally irrelevant*. A square randomly appeared in one of the eight possible FoE locations (the *prime* or *cue*) for 1 s, and disappeared before the optic flow display onset. A delay of 150–300 ms was introduced between the prime and optic flow presentations. Instead of being instructed to saccade to the location of the FoE, the monkeys were trained to saccade to the location of the prime, irrespective of the FoE location. Due to the random positioning of the prime, it could have occupied the three nearest (*near condition*) or farthest (*far condition*) positions relative to the FoE during the trial. The near and far conditions therefore constituted subsets of the behaviorally irrelevant trials. Monkeys saccaded to the prime location more than a second after the prime disappeared, hence it is assumed that some attentional signal was maintained on the prime location until the trial concluded. Behaviorally relevant trials did not include a prime, but it is assumed that the monkeys attended the centrally located fixation point. The monkey had no information to predict where the FoE would appear. We simulated the two experimental conditions of Dubin and Duffy (2007, 2009): behaviorally relevant and behaviorally irrelevant trials.

Dubin and Duffy analyzed neuronal tuning curves as a function of FoE location, and the average firing rates of neurons over time across the trials were derived for subpopulations of cells that showed statistically significant effects for the behaviorally relevant and behaviorally irrelevant trials (near and far). Significance was assessed using analysis of variance (ANOVA) with Greenhouse-Greyser correction for non-spherical variance. Dubin and Duffy recorded from 135 MSTd neurons in total, and 32 cells showed significant effects to conditions assessed in the study. With respect to experimental condition (behaviorally relevant and irrelevant), 16 exhibited significant effects for a single condition, 6 showed significant effects to both conditions, and 6 showed a task by FoE location effect (28 cells total). With respect to the relative position between the FoE and the prime, 11 cells exhibited significant effects for near vs. far, 4 for behaviorally relevant vs. irrelevant task types, 1 for both types, and 6 for task by FoE location (22 cells total).

**Figure 2:**
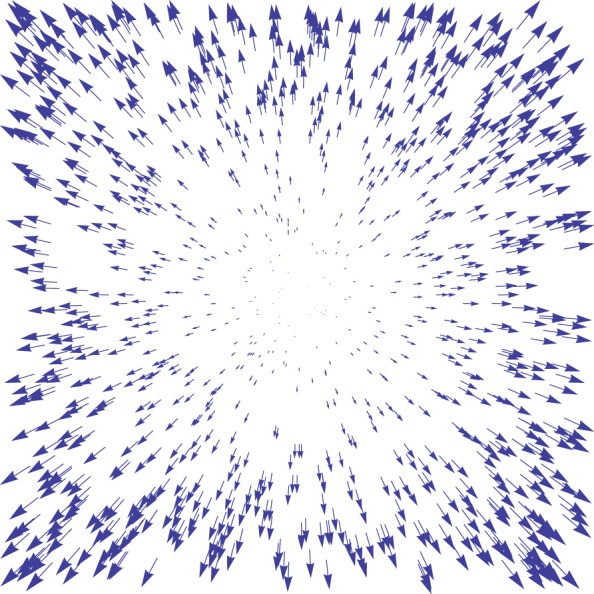
**A sample optic flow field used in the model simulations.** The base of each arrow represents the instantaneous position of one of 1000 dots, the direction indicates the dot's instantaneous direction of travel, and the arrow length is proportional to the speed.

In our simulations, we generated 1000 dot radial optic flow displays that occupied 256 × 256 pixels, as shown in Figure 2. Following the protocol of Dubin and Duffy, we constrained the dot movement speed to follow a cos(θ)sin(θ) function, where θ denotes the visual angle between the observer's gaze or line of sight on the FoE and each dot position located 1 m in depth (Duffy and Wurtz, 1991). After adjusting the speed of each dot according to this formula, we scaled the dot speeds such that the average velocity of all the dots over the entire trial was ~40°/s. Our results were the same whether we constrained dots to move at an average fixed speed (~40°/s) as Dubin and Duffy did or simulated random dots observed at a walking speed toward a simulated fronto-parallel plane 1 m in depth. We simulated the dynamics of 128 MSTd neurons, each having a receptive field centered on equally spaced positions along the middle horizontal axis of the display. Each MSTd unit had a large 90° × 90° receptive field, consistent with neurophysiological data (Duffy and Wurtz, 1995). To analyze the model MSTd population results, we averaged the activity of all neurons over 500 ms following the onset of the optic flow display. For simplicity, we simulated FoE locations only along the center horizontal axis. In order to derive neuronal tuning curves, we selected 10 neurons tuned to similar FoE and analyzed their responses to behaviorally relevant and behaviorally irrelevant trials whereby the optic flow field FoE occupied one of nine evenly spaced locations along the 90° middle horizontal axis. Our selection of 10 neurons is comparable to the 11 used by Dubin and Duffy. For each FoE location and trial type, we averaged the neurons' activity 50–250 ms after the onset on the optic flow display.

In the behaviorally relevant condition the monkeys could not anticipate the FoE location and in the behaviorally irrelevant condition the prime disappeared over a second before the monkey had to saccade. We find it unlikely that the difference in results can be attributed to residual bottom–up activity. We therefore assume that the monkeys attended the prime position between the time it appeared and when the saccade was made. Some spatial (attentional) neural signal indicated whether the monkey should saccade to either the prime or FoE location. In our behaviorally relevant trials (without a prime), we simulated a spatial distribution that enhanced the activity of neural units sampling the FoE position. By contrast, in the behaviorally irrelevant condition, the activity of neural units sampling the prime location were enhanced. We modeled the experimental conditions by adjusting the spatial position of a Gaussian-distributed neural signal originating from FEF. Microstimulation of FEF has been shown to locally modulate the contrast sensitivity of cells in areas such as V4 (Reynolds and Chelazzi, 2004).

(1) $$G(x;c,\mu ,\sigma^{2}):=\frac{c}{\sqrt{2\pi \sigma^{2}}}e^{\frac{-{\left(x-\mu \right)}^{2}}{2\sigma^{2}}}$$

Equation (1) specifies the Gaussian distribution that was sampled to derive the spatial profiles of the neural signals from model areas FEF to MSTd. In the behaviorally relevant condition, we set μ such that *G* was centered on the fixation point, since the monkey was trained to saccade to the radial center of the optic flow located 30° away. In the near and far conditions, we set μ such that the Gaussian was centered 43% and 86% closer to the edge of the screen away from the FoE position. This simulates the 30° and 60° radial distance between FoE and prime locations used by Dubin and Duffy in their near and far conditions. We configured the Gaussian distribution to wrap around the population boundaries such that the area under the curve for all conditions remained constant. Across these three conditions, we fixed *c* = 6.5 and σ = 18.5°. In the experiments of Dubin and Duffy (2009), the behaviorally irrelevant trials encompassed both near and far conditions. To simulate the behaviorally irrelevant condition, we averaged across all trials with different prime locations (near and far). To investigate whether or not the type of MSTd attentional modulation is important, we applied the spatial signal from FEF in three different ways: by multiplying or adding the signal to the input of MSTd, and by modulating the gain of MSTd sensory inputs. Adding the attentional signal to the sensory input that MSTd units receive can shift their receptive fields, which approximates the behavior of non-multiplicative attention (Womelsdorf et al., 2006). Multiplying the FEF signal with the sensory input MSTd units receive enhances or suppresses existing activity, which approximates the behavior of multiplicative attention (Martinez et al., 1999). To ensure our model tested a variety of attention types, we multiplied the model FEF signal to the MSTd sensory input such to only augment the gain of existing neural activity. In other words, units influenced by attention in this case exhibit higher gain in their activations than those that are not.

### The model

As noted in the Introduction, the model contains stages that correspond to primate visual areas V1, MT, MSTd, and FEF. Model V1 projects to MT, which provides bottom–up inputs to MSTd. FEF provides top–down attentional signals to MSTd.

All simulations were run on a 8-core 2.66 Ghz Mac Pro with 64 GB of memory using Mathematica 8. Parameter values listed in the text specify those that remained constant throughout all simulations. Table 1 contains parameters values that varied for different experimental conditions.

**Table 1:** **Parameter values used in simulations**.

**Attention case**	**γ**_**MST**_	**δ**	***n***	**ζ**	***w***_**0**_
Non-multiplicative	0.03	1	3	0.0001	0.15
Multiplicative	0.014	1	3	4×10^−14^	0
Multiplicative gain	0.03	1	3	0.0005	0
Modified sigmoid	0	1.2	6	3×10^−9^	0.08
Modified sigmoid (windowed)	0	1.1	6	2×10^−9^	0.065
Modified sigmoid (distance-dependent)	0	1	6	4.5×10^−9^	0.057

Equations in our model describe the temporal dynamics of individual neurons or populations of neurons that densely sample the visual field. Model neurons obey ordinary differential equations that feature *shunting* competitive dynamics (Grossberg, 1968). These equations perform a leaky integration of their inputs and simulate many known properties of neurons, such as divisive normalization (Heeger, 1992; Carandini and Heeger, 2011) and automatic gain control (Grossberg, 1983). Model equations for area MSTd resemble the following membrane equation studied by Grossberg (1973), termed a *recurrent competitive field*:

(2) $$\frac{dx_{i}}{dt}=-\alpha x_{i}+(\beta -x_{i})(f(x_{i})+I_{i})-(x_{i}+\gamma )\sum_{k\neq i}f(x_{k}).$$

Equation (2) is a shunting equation that describes the activity, *x*, of the *i*th cell in a neural network layer. The parameters α, β, and γ define the passive decay rate (s^−1^), saturation upper bound, and hyperpolarizing lower bound of the cell, respectively. The terms (β − *x*_*i*_)(*f*(*x*_*i*_) + *I*_*i*_) and −(*x*_*i*_ + γ)∑_*k* ≠ *i*_ *f*(*x*_*k*_) of Equation (2) specify the shunting excitation by input *I* and surround inhibition, respectively. In Equation (2), *f*(*x*) is a *signal function* (Grossberg, 1973) that specifies the nature of the feedback from cells in the same network layer. A sigmoidal signal function, Equation (3), induces winner-take-all, pattern-preserving, and uniformizing behavior when the activation of units in the model falls in the faster-than-linear, linear, and slower-than-linear regions of the signal function, respectively. For a more comprehensive analysis of recurrent competitive fields, such as those defined in Equations (2) and (9), see Grossberg (1973). The parameters δ, *w*_0_, ζ, and *n* adjust the gain, threshold, slope and position of the linear portion, and slope of the sigmoid, respectively. In Equation (3), [·]^+^ denotes half-wave rectification, max(·, 0).

(3) $$f(w;\delta ,w_{0},\zeta ,n)=\frac{\delta {\left({\left[w-w_{0}\right]}^{+}\right)}^{n}}{\zeta +{\left({\left[w-w_{0}\right]}^{+}\right)}^{n}}$$

### V1

In model V1, we analytically compute first-order optic flow field representations according to Equation (4) (Longuet-Higgins and Prazdny, 1980), to create motion representations similar to those shown in Figure 2.

(4) $$I^{t}(x,y):=\left(\begin{array}{c} \dot{x}\\ \dot{y} \end{array}\right)=\frac{1}{Z}\left(\begin{array}{c} xl_{z}-l_{x}\\ yl_{z} \end{array}\right).$$

In Equation (4), (*ẋ*, *ẏ*) represent the horizontal and vertical flow components at the position (*x*, *y*) at time *t*, *l*_*z*_ signifies the depth component of the translational velocity of the observer (m/s), *l*_*x*_ indicates the horizontal component of the observer translation (m/s), *Z* is the distance (m) from the observer to the point in space represented by the dot. We use the notation *I*^*t*^(*x*, *y*) to represent the vector-valued optic flow field (*ẋ*, *ẏ*) with spatial location (*x*, *y*) at time *t*.

### Model MT

Model V1 projects directly to Model MT, where cells have receptive fields that integrate over particular velocities (speed and direction). We define the pooled MT motion $\vec{M}(x,y)$ according to

(5) $$\frac{d\vec{M}(x,y)}{dt}=-\alpha_{\text{MT}}\vec{M}(x,y)+(I^{t}(x,y)*G_{\text{MT}})(x,y;\mu_{\text{MT}},\Sigma_{\text{MT}},r_{\text{MT}})$$

where * denotes the 2D convolution operator, α_MT_ represents the passive decay rate (s^−1^) of each MT component unit, *G*_MT_ is a 2D discrete multivariate Gaussian kernel with mean μ_MT_ and covariance matrix Σ_MT_ normalized such that all points in the kernel's support sum to unity, and *r*_MT_ defines the kernel radius (°). The points (*x*, *y*) refer to positions in 2D retinotopic coordinates. For all simulations, we set α_MT_ = 3. We model MT cells with circular receptive fields, hence we set $\mu =\vec{0}$ and Σ_MT_ such that σ_*x*_ = σ_*y*_ = σ_MT_ and the covariance ρ between *x* and *y* is zero. We used σ_MT_ = 0.01° and *r*_MT_ = 3° to conservatively simulate MT cell receptive field properties found in neurophysiological studies (Born and Bradley, 2005; Churchland et al., 2005). Rather than integrating each component of Equation (5), we used the analytical solution shown in Equation (6) to evaluate each model MT cell at time *t*.

(6) $${\vec{M}}^{t}(x,y)=\frac{I^{t}(x,y)}{\alpha }*G_{\text{MT}}(x,y)(1-e^{-\alpha t})$$

### Model MSTd

In model MSTd, we perform a template match between MT units ${\vec{M}}^{t}(x,y)$ and all templates ${\vec{T}}_{i}(x,y)$. That is, for a given ${\vec{M}}^{t}(x,y)$, we match against ${\vec{T}}_{i}(x,y)$ for all horziontal headings *i*. Templates are defined as normalized radial optic flow fields computed according to Equation (4) (Layton et al., 2012). We used 128 templates, each with a different FoE position uniformly sampled along the 90° middle horizontal axis. We weight the template match using inverse Euclidean distance. In Equation (7), we obtain a scalar value *p*^*t*^_*i*_ for time *t* at the horizontal heading *i*, representing the cosine similarity (i.e., inner product) between distance-weighted vectors at each time and those in the template.

(7) $$p_{i}^{t}=\lambda \sum_{\left\{x,y\right\}}W_{i}(x,y)\left(\sum_{\left\{\dot{x},\dot{y}\right\}}\frac{{\vec{T}}_{i}(x,y)⊙{\vec{M}}^{t}(x,y)}{\left||{\vec{M}}^{t}(x,y)|\right|}\right)$$

In Equation (7), *W*_*i*_(*x*, *y*) represents a distance-dependent weighting from the horizontal heading indexed *i*. We use inverse 2D Euclidean distance, scaled by a parameter λ to adjust the effective spatial extent of the templates. We selected λ = 200, which broadly scales the distance-dependent weights across the visual field. The inner summation performs component-wise multiplication (denoted by the ⊙ operator) between vectors in the template ${\vec{T}}_{i}(x,y)$ and on MT ${\vec{M}}^{t}(x,y)$ for every spatial location. The resulting vector is normalized by the *L*^2^ (Euclidean) norm (denoted $\left\|{\vec{M}}^{t}(x,y)\right\|$) and then the vector components {*ẋ*, *ẏ*} are summed.

We subsequently smoothed then sharpened the 1D pattern match distribution in MSTd according to

(8) $$P_{i}^{t}:={\left(p^{t}*G_{\text{MST}}\right)}^{n_{\text{MST}}}(i;\mu_{\text{MST}},\sigma_{\text{MST}},r_{\text{MST}})$$

where * is 1D cyclic convolution and *G*_MST_ is a normalized 1D Gaussian kernel. We set the radius *r*_MST_ to 40°, σ_MST_ = 10°, and mean of the MST kernel $G_{\text{MST}}\;\mu_{\text{MST}}=\vec{0}$. We sharpened the resulting distribution with *n*_MST_ = 30.

Finally, we introduce a dynamical competitive network to describe each MSTd unit *B* at location *i* according to Equation (9).

(9) $$\frac{dB_{i}}{dt}=-\alpha_{\text{MST}}B_{i}+(\beta_{\text{MST}}-B_{i})(f(B_{i})+P_{i}^{t})-(\gamma_{\text{MST}}+B_{i})\sum_{k\neq i}f(B_{k}).$$

In Equation (9), *f*(*B*_*i*_) and ∑_*k* ≠ *i*_ *f*(*B*_*k*_) represent excitatory and inhibitory recurrent inputs, respectively. The self-excitation term *f*(*B*_*i*_) can be considered as a form of post-synaptic input if one makes an assumption that the cell does not have a physical synaptic connection with itself. We set α_MST_ = 0.01 (s^−1^) and β_MST_ = 1.

### Model FEF

In Dubin and Duffy (2007, 2009) attention was maintained prior to the optic flow presentation. We modeled the FEF signal as decaying (Equation 10) after the initial conditions were set according to Equation (1) at *t* = 0. The parameters varied according to the experimental conditions defined above. We set α_FEF_ = 0.01 (s^−1^).

(10) $$\frac{dA_{i}}{dt}=-\alpha_{\text{FEF}}A_{i}.$$

Modulation of neuronal activity due to attention has been documented in visual areas fewer synapses away from the retina than MSTd, such as V1 (Paradiso, 2002). This article is focused on the response properties of MSTd as described by Dubin and Duffy, we therefore implemented attentional effects only as far as MSTd and investigated whether the effects of attentional signals on the MSTd population may be sufficient to explain the data.

### Attention cases

Exactly how attention acts in cortex is not clear, but researchers have proposed that it could either multiplicatively or non-multiplicatively affect neural signals. We tested several possible ways an attentional signal could interact with MSTd cells. First, we considered *additive attention* on MSTd cells by changing the excitatory (2nd) term in Equation (9) to (β_MST_ − *B*_*i*_)(*f*(*B*_*i*_) + *P*^*t*^_*i*_ + *A*_*i*_), where *A*_*i*_ is defined according to model FEF (Equation 10). This modification provides an additive (non-multiplicative) influence on MSTd unit inputs (Womelsdorf et al., 2008). Second, we considered the effects of *multiplicative attention* on MSTd dynamics by modifying the excitatory term in Equation (9) to (β_MST_ − *B*_*i*_)(*f*(*B*_*i*_) + *P*^*t*^_*i*_ × *A*_*i*_). In this case, MSTd activity is enhanced or suppressed but not induced (Martinez-Trujillo and Treue, 2004). We also examined the effects of *multiplicative gain* by scaling the sensory input *P*^*t*^_*i*_ in Equation (9) by the spatial pattern from FEF, which modulates the gain of model cell responses: (β_MST_ − *B*_*i*_)(*f*(*B*_*i*_) + *P*^*t*^_*i*_ × (*A*_*i*_ + 1)).

## Results

### MSTd population timing

**Figure 3:**
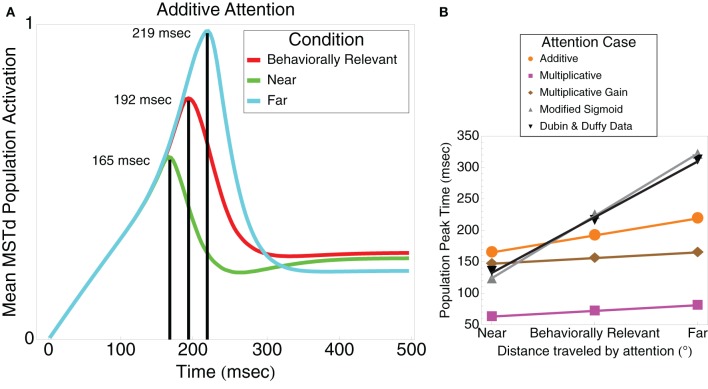
**(A)** The model mean MSTd population activation yields a linear (*R*^2^ = 1) separation between the peak timings in the near (165 ms), behaviorally relevant (192 ms), and far (219 ms) conditions, respectively. In these respective conditions, the model simulates the increasing distance attention traveled between the fixation and FoE locations. **(B)** Summary of the population peak timing in the near, behaviorally relevant, and far conditions as a function of the type of attention modeled. Additive, multiplicative, and multiplicative gain attention types all produced a linear peak timing (*R*^2^ > 0.98). Modifying the sigmoidal signal function in model MSTd, as described in the text, yielded the best timing correspondence to the Dubin and Duffy (2009) data (gray line).

In order to assess the model's ability to fit the basic neuronal tuning curve and linear trend in the peak population temporal activity of Dubin and Duffy (2007) and Dubin and Duffy (2009), we employ the sigmoidal signal function defined by Equation (3) with *n* = 3. Figure 3B summarizes the peak timing results as a function of attention. As noted, we simulated non-multiplicative (additive), multiplicative, and multiplicative gain attention conditions. All attention simulation results exhibit a linear peak timing trend similar to that found in the data of Dubin and Duffy (2009) (Figure 1). Figure 3A shows the mean MSTd population response in the additive attention case. The timing of the MSTd population activity peaks are at 165, 192, and 219 ms (*R*^2^ = 1) corresponding to when the prime is at 0° eccentricity (near condition), the FoE is at 30° eccentricity (behaviorally relevant condition), and the prime is at 60° eccentricity (far condition). In this case, the peak latencies exhibit an exact linear trend. In the multiplicative attention case, we obtained MSTd population peak latencies of 63, 72, and 81 ms for the near, behaviorally relevant, and far conditions, respectively. In the multiplicative gain case, we obtained MSTd population peak latencies of 147, 156, and 165 ms for the near, behaviorally relevant, and far conditions, respectively. We obtained linear peak timings (*R*^2^ > 0.98 in all conditions) and similar qualitative appearances irrespective of the attention condition (Figure 3B). For the remainder of this article we focus on the additive case.

**Figure 4:**
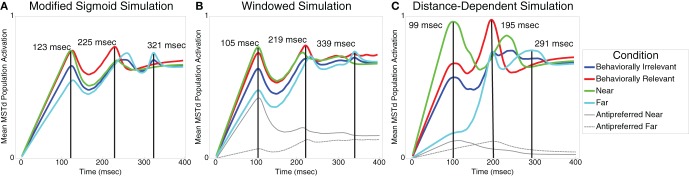
**(A)** Model mean MSTd population activation using a steeper sigmoidal signal function compared to Figure 3A (see Table 1). The model produces linear peak timing in near (green), behaviorally relevant (red), and far (cyan) conditions while qualitatively simulating the low frequency deflections present in the Dubin and Duffy (2009) data (Figure 1). The behaviorally irrelevant condition (blue) does not yield a distinct peak, thereby qualitatively matching the data of Dubin and Duffy (2009). **(B)** Model mean population MSTd population activation of cells selective to the FoE position (windowed). The solid and dashed gray curves show the activation of units not selective to the FoE in the near and far conditions, respectively. **(C)** Model mean MSTd population activation in a network that features distance-dependent competition and windowing, which better reflect neurophysiological and experimental conditions. Consistent with the data of Dubin and Duffy (2009) (Figure 1), the curves for all conditions drop off more steeply after peaking, the peak in the behaviorally relevant case has higher contrast, and the behaviorally irrelevant condition response is flatter.

When we modified the MSTd network signal function (“Modified Sigmoid”) as specified in Table 1, we were able to better model the temporal dynamics of the MSTd neurons and obtain closer correspondence to the Dubin and Duffy (2009) data (Figure 3B). Changing the sigmoid exponent *n* from 3 to 6 steepened the slope of the sigmoid. The implications of this change are that the activity of network units is less likely to enter the linear range of the sigmoid signal function because it is narrower, and units whose activity do enter the linear range are more likely to be “pushed” out to the faster-than-linear or slower-than-linear segments. Figure 4A depicts the average population behavior over time with the new signal function. The timing of the MSTd population activity peaks are at 123, 225, and 321 ms corresponding to when the prime is at 0° eccentricity (near condition), the FoE is at 30° eccentricity (behaviorally relevant condition), and the prime is at 60° eccentricity (far condition). Similar to the data of Dubin and Duffy (2009), the behaviorally irrelevant population response (blue) did not yield a dominant peak. The modified signal function better fits the data of Dubin and Duffy (2009). We verified the network was making use of the full dynamic range of the sigmoidal signal function by comparing the network behavior to when we made *f*(*w*) a step function with threshold Γ = 0.25 to emulate the shape of the sigmoid without the linear portion. The step signal function preserved the linear peak timings, but did not yield the multiple deflections seen in Figure 4A, which indicates that model MSTd units use the linear region. The modified signal function gives rise to a number of low frequency deflections in the population activity that resemble those present in the Dubin and Duffy data (Figure 1). Our simulations did not contain noise, so we investigated the source of these deflections.

**Figure 5:**
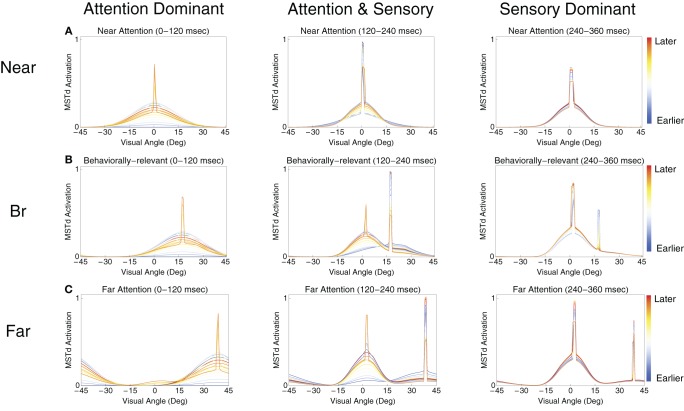
**The spatial dynamics of the model MSTd heading template match layer. (A–C)** Show the temporal evolution of MSTd cells in the near, behaviorally-relevant, and far conditions, respectively. Each subplot contains snapshots of the network activity at uniformly sampled times within 120 ms intervals. From left to right, the plots show snapshots from contiguous 120 ms intervals. The FoE is located in the center of the visual display (0°). We call the cells primarily influenced by the sensory optic flow (left peak) and attentional (right peak) signals the *sensory subpopulation* and *attentional subpopulation*, respectively.

Figure 5 presents snapshots of the spatial activity in the template matching layer for the near, behaviorally relevant, and far conditions. Each subplot shows 120 ms of network activity within model area MSTd. We used intervals of 120 ms to capture the dynamics around the low frequency deflections present in Figure 4. In each set of plots, we identified a group of MSTd units that respond primarily to the attentional signal (the *attentional subpopulation*) and another group primarily driven by the sensory bottom–up input (the *sensory subpopulation*). Note, the subpopulations are in the same model MSTd layer and are only classified as such based on their response to either the sensory optic flow or attentional signal. The results in Figure 5 indicate that the deflections arise due to competitive interactions between the attentional (e.g., Figure 5C, right peak) and sensory (e.g., Figure 5C, left peak) subpopulations. Both attentional and sensory inputs influence model neurons within each subpopulation, so we use the terms attentional and sensory subpopulations to refer to the primary distributions of activity within the network induced by the respective signals. In the near condition (Figure 5A), the first deflection, which happens to be the overall population peak activity, occurs due to the superposition of the MSTd response driven by the attentional signal (built up prior to the arrival of the sensory input) and the sensory input due to the optic flow (activity still ramping up at ~120 ms) (Figure 5A, left panel). Due to the competition, the average activity across the network subsequently reduces for the next ~50 ms. Finally, the MSTd response to the sensory input reaches its peak at ~225 ms and then reduces due to competition at later times (Figure 5A, center and right panels). In the behaviorally-relevant condition, covert attention must travel 30° relative to fixation. Unlike the near condition, the sensory and attentional signal superposition is weaker when the first deflection occurs at ~120 ms (Figure 5B, left panel). However, the superposition is stronger later, resulting in the second deflection at 225 ms when attention reinforces the bottom–up response to the optic flow (Figure 5B, center panel). In the far condition, the prime is located at 60° eccentricity relative to the FoE. The first deflection corresponds to the high MSTd activity due to the attentional subpopulation (Figure 5C, left panel). Since the sensory response is still developing in MSTd and the distance is far from the prime, less superposition occurs and the network response is more evenly distributed across MSTd, resulting in a lower average population activity than the other experimental conditions. The second deflection in the far condition arises due to fierce competition between the sensory and attentional subpopulations (Figure 5C, center panel). The attentional subpopulation loses the competition due to the emerging sensory response, which results in a sudden dip in the average network activity at ~300 ms. Finally, the third deflection at 310 ms corresponds to the sensory subpopulation reaching its maximal activity and the rapid decay of the attentional subpopulation (Figure 5C, right panel). The sensory subpopulation response takes longer to develop, because of the strong competition.

### Windowing

Dubin and Duffy included only neurons that showed significant responses to the experimental conditions in their analyses. We initially included all model cells. In order to better approximate electrophysiological conditions, we introduced windowing whereby we only included cells moderately or highly selective to the FoE of the optic flow display. This way, we do not include cells that would not elicit a response to the sensory optic flow in our averaging. This was the criterion employed by Dubin and Duffy (2009) to select neurons for analyses. It should be noted that this affords a better data fit, but does not qualitatively affect our results. The simulation results are shown in Figure 4B. Figure 4B shows that peak latencies were 105, 219, and 339 ms for the near, behaviorally relevant, and far conditions, respectively. The behaviorally irrelevant condition (blue) did not yield a clear peak. We plotted the activations of units excluded from the colored curve averages in the near and far conditions in solid and dashed gray, respectively. Similar to the Dubin and Duffy (2009) data, the gray curves resided below the colored curve conditions, and the solid gray curve showed higher average activation than that of the dashed gray curve.

### Distance-dependent competition

Our model assumes all cells globally compete with one another with equal weight, despite the fact that they may have very different visuotopic preferred FoE locations. Since MST has a rough topography (Born and Bradley, 2005), we introduce a distance-dependent weighting in Equation (9) such that units compete locally. Each MSTd unit now receives bottom–up input from a single visuotopic location but receives only local inhibitory input from neighboring units. The extent of the local competition in MSTd is determined by the inhibitory kernel *G*_MST_. As shown in Figure 4C, the distance-dependent network results combined with windowing further improves the qualitative fit with the Dubin and Duffy (2009) data. The smaller peak in the near condition appears after the second deflection in the far condition, the contrast between the first two behaviorally relevant peaks is higher, and the mean MSTd population activity drops off faster after the peaks occur in all conditions. Peak latencies were 99, 195, and 291 ms for the near, behaviorally relevant, and far conditions, respectively.

(11) $$\frac{dB_{i}}{dt}=-\alpha_{\text{MST}}B_{i}+(\beta_{\text{MST}}-B_{i})(f(B_{i})+P_{i}^{t})-(\gamma_{\text{MST}}+B_{i})\sum_{k\neq i}\sum_{l}G_{\text{MST}}(k-l,\sigma^{2})f(B_{l}).$$

Equation (11) shows the modified MSTd equation to implement the distance-dependent interactions via *G*_MST_(μ_MST_, σ^2^_MST_), with *c*_MST_ = 4, σ = 60°. Simulation results are shown in Figure 4C. Compared to the results shown in Figures 4A,B, the attentional and sensory subpopulations are composed of fewer units due to the local inhibition, which results in larger drops in average network activity when the sensory representation wins over the attentional representation in the competition.

### MSTd neuronal tuning curves

**Figure 6:**
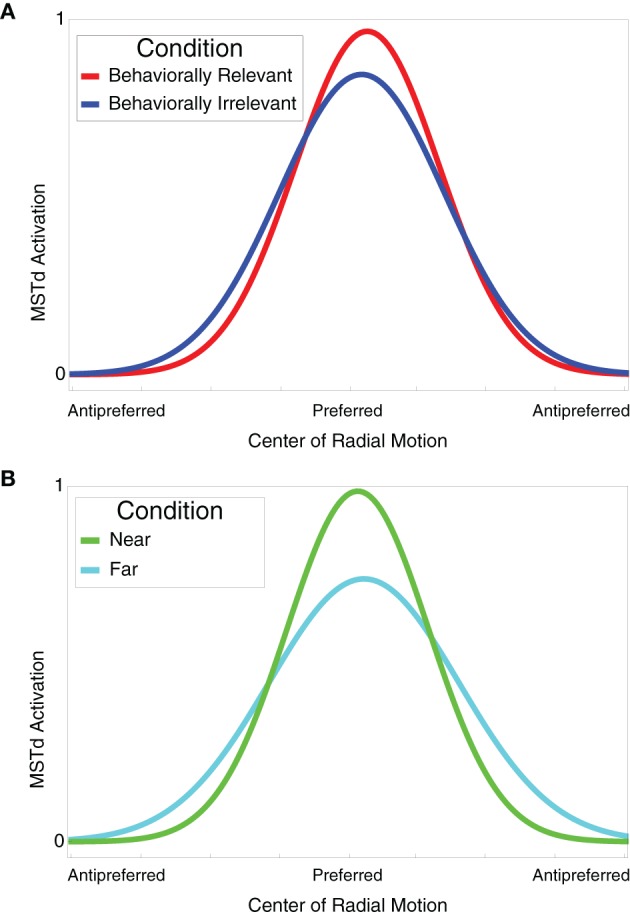
**(A)** Behaviorally relevant (red) vs. behaviorally irrelevant (blue) attention tuning curves. Units selective to radial center of motion exhibited higher grain in the behaviorally relevant condition compared to the behaviorally irrelevant condition. Optic flow displays with FoE far from the preferred location elicited a suppressed response in units. **(B)** Near (green) vs. far (cyan) attention tuning curves. Units selective to radial center of motion exhibited higher grain in the near condition compared to the far condition. Optic flow displays with FoE far from the preferred location elicited a suppressed response in units. The tuning curves were derived from 10 model cells.

Figure 6 shows the tuning curves of model neurons selective to similar optic flow FoE in the near vs. far (Figure 6B) and behaviorally relevant vs. behaviorally irrelevant (Figure 6A) conditions. When presenting an optic flow display at MSTd units' preferred FoE location, units exhibited a higher gain in the near and behaviorally relevant conditions compared to far and behaviorally irrelevant conditions, respectively. When the units were not tuned to the FoE position, units in these respective conditions showed suppression. The tuning curves derived in Figure 6 reflect the experimental findings of Dubin and Duffy (2007).

## Discussion

We have presented a dynamical model of primate MSTd that simulates the data of Dubin and Duffy (2007, 2009). Our model produced peaks in the average population activity of MSTd units with timings spaced linearly as the distance increased between the center of the spatial attention signal and the FoE. The linear trend in the peak timings was robust to a range of parameters, including the steepness of the sigmoidal signal function slope and signal function type (e.g., faster-than-linear and step functions). Our quantitative fits of the data of Dubin and Duffy (2007, 2009) improved when we excluded units from the population average that were not strongly selective for the optic flow FoE and introduced distance-dependent competition into the network. Both modifications more accurately simulate the electrophysiological and neurophysiological conditions of Dubin and Duffy (2007, 2009). Our neuronal tuning curves exhibited increased gain about the neuron's preferred FoE when comparing the curves generated from the aggregate behaviorally relevant vs. behaviorally irrelevant conditions and the near vs. far conditions. These findings also match the data of Dubin and Duffy (2007, 2009). Finally, we simulated multiplicative and non-multiplicative types of attention acting on model area MSTd, and our model produced qualitatively similar results irrespective of the particular type of attention. Hence, our model is agnostic with respect to, and compatible with all of these forms of attention. Our MSTd network equations feature built-in normalization (Equation 9), and are also compatible with proposals of attention that incorporate a normalization property (Reynolds and Heeger, 2009; Carandini and Heeger, 2011).

The primary focus in selecting our model structure and parameters (Table 1) was to use the minimal possible mechanisms to fit the linear peak shift latencies. We tested three different attentional mechanisms by adding as few parameters the model as possible, while still capturing the expected behavior of each attention type. Relative curve separations in the data (Figure 1) and our model (Figure 4) agree well quantitatively at different temporal milestones. Early in the “Windowed” and “Distance-dependent” simulations at ~100 ms, the ratio between the green curve peak height and the heights of the cyan, purple, and red curves underneath are close to those in the data. Late in all the simulations, the ratio between heights of the cyan curve peak and the green curve is also close to the data. However, in some cases the ordinal heights of the curves in simulations differed from that of the data. For example, under the cyan curve peak in the data, the green curve was the next highest, followed by the red and purple curves. In the “Distant-dependent simulations,” the secondary green curve peak that occurs after 200 ms decreases faster than it does in the data and therefore it is the least active at the time of the cyan curve peak. Conversely, the behaviorally irrelevant condition curve decreases more slowly than it does in the data and therefore is higher than the red curve. There are several reasons why this may occur. First, an exhaustive search through the model parameter space has not been performed, so parameters that improve fits to the relative separations between experimental curves may exist. Second, the model fits the average neural data from Dubin and Duffy and does not consider the variance. Third, many known properties of MSTd cells are not modeled, such as differential receptive field sizes (Duffy and Wurtz, 1991), dynamic ranges, and speed tunings (Duffy and Wurtz, 1997). Our goal was identify the simplest core neural mechanisms that are required to give rise to the data of Dubin and Duffy (2007, 2009).

The exact implementation of attention (additive, multiplicative, and multiplicative gain) in the model did not affect the linear separation in the peak latencies, but the fact that the model attentional signal was concentrated in particular spatial locations and assumed a Gaussian-like form is crucial for the model's ability to fit the data. In particular, the attentional signal modulated the recurrent inputs in Equation (9), which resulted in higher firing rate amplification closer the FoE, despite the attentional signal's exact form, and therefore decreased the mean MSTd population activity peak latency when the attentional signal acted more proximally to the FoE location. Note that if the recurrent competition is not included in the model, the population peak activity will always occur at the same time, defined by the temporal, not the spatial, separation of the attentional and sensory signals. Without the recurrent competition components, Equation (9) becomes a leaky integrator and the absolute magnitude of the input signals is the same in each experimental condition. With windowing, the effect of additional stimulation from the attentional signal on the FoE sensitive population varies depending on the spatial position of the attentional prime, however, unless a subset of MSTd cells saturate, these differences will have only minimal effects on the timing of the peak population response. Recurrent competition prolongs the time that the attentional signal produces a high activation response across the cell population after the attentional prime is removed. This latent activation remains until there is a competing sensory signal. When the latent activation peak is close to the sensory activation peak the two populations merge to produce an overall population peak soon after the presentation of the sensory signal. When the latent activation peak is far from the sensory activation peak, the two populations compete. Initially the latent activation is able to suppress the sensory activation, but as the competition continues the sensory activation overtakes and suppresses the latent activation. In this case, the overall population peak occurs when uncertainty as to the winner is at its highest, in other words when the latent activation and the sensory activation are roughly equal. We predict that attentional signals from FEF act on target MSTd neurons that possess a FoE preference near the spatially primed location of the visual field and receive the most modulation. Neurons with distal FoE preferences are predicted to successively receive less modulation as a function of visuotopic distance. Our model would require revision if a future experiment demonstrated that saccade-related attentional signals from FEF *do not* mostly target MSTd neurons with FoE preferences spatially coincident with the primed location or if the signal targets MSTd neurons in an erratic rather than a visuotopic, Gaussian-like fashion. In our model simulations, we assumed that attention modulates neurons in MSTd prior to sensory signals. This assumption is based on a recent study of saccadic planning, which reports that saccade-related neural activity arrives 30 ms earlier in MSTd than would otherwise occur without saccades during fixation (Crowder et al., 2009). Future studies that probe the difference in latencies between MSTd afferents from MT and FEF could quantitatively test the prediction made by the model that attentional signals arrives prior to sensory signals, perhaps by simultaneously recording from neurons in MSTd and FEF. Experiments could also investigate the duration of FEF modulation on MSTd neurons when monkeys are presented with optic flow to test whether the signal influence decreases over time as predicted by the model.

As noted, the recurrent inputs to MSTd units in Equation (9) played an important role in fitting the data. Recurrent excitation and inhibition in model MSTd is modulated by a sigmoidal signal function (*f*(*w*) in Equation (9)). While we were able to obtain evenly spaced peak separations, akin to those shown in Figure 3, with a faster-than-linear signal function, such as *f*(*x*) = *x*^2^, we obtained the best performance using a sigmoid function. Depending on a unit's activity relative to the spatial pattern of activity in the network, sigmoid signal functions afford analog winner-take-all behavior in the network. Units whose activity falls in the slower-than-linear portion of the sigmoidal signal function apply pressure on the rest of the population to suppress lower activity maxima, which in turn further enhances the global activity maximum. Unlike prior analysis of recurrent competitive fields with sigmoid signal functions (Grossberg, 1973), our MSTd simulations have dynamic, continuously varying inputs, which directly impact the recurrent feedback to MSTd units. Due to the continuously changing inputs and the use of a sigmoid signal function, strong bottom–up inputs can override stable network patterns, such as in the case when the attention signal is the main MSTd input, and apply pressure on the MSTd population to shift the location of the globally most active unit. This interplay between bottom–up, top–down, and recurrent inputs gives rise to the activity patterns shown in Figure 5.

Recurrent competition, FoE selective cells in MSTd, and temporal competitive dynamics represent essential characteristics of the model. We know of no other model that can fit the data of Dubin and Duffy (2007, 2009). Balancing rising MT unit activation and decaying attentional signals from FEF in the model allows competition within MSTd to produce the distinct activity peaks observed in Figure 5, which correspond to temporal landmarks that indicate that a particular signal is winning the competition. The dynamical properties of the MT and FEF signals represent important aspects of the model that allows the mean population activity in MSTd to peak at the appropriate times. A prior version of the model cannot simulate this because it employs difference equations (Layton et al., 2012). Other models that lack temporal integration (Royden, 1997; Raudies et al., 2011), that are filter-based (Perrone, 1992), or that lack continuous-time dynamics (Browning, 2012), lack the necessary mechanisms to balance bottom–up and top–down signals and are therefore unlikely to be able to simulate the timing of peaks in the Dubin and Duffy data. Because our model does not include spiking, synaptic and conduction delays, temporal dynamics at the neuronal level may differ compared to those implemented in the model. Further experiments that show finer grain timing details about how attentional signals interact with MSTd neurons may require the model to be modified.

Our quantitative fits to each condition in the data of Dubin and Duffy (2007, 2009) were achieved by the increased distance between the attentional and sensory signals defined in the experimental paradigm. All model parameters were fixed, except where explicitly noted in the text. The differences in peak timings across the behaviorally relevant, near, and far experimental conditions arise in the simulation due to the competitive interactions between attentional and sensory representations in MSTd. Although the low frequency deflections in the data of Dubin and Duffy (2009) may appear to reflect random fluctuations, we simulated these properties of the MSTd population response without any noise (Figure 4). We therefore predict that the low frequency deflections present in the neurophysiological data may represent dynamic shifts between the sensory and attentional subpopulations in MSTd. Figure 5 shows how the increased distance between the attentional and sensory signals result in fiercer competitive interactions and oscillations between the MSTd subpopulations. The temporal integration of the recurrent competition and sigmoidal signal function were critical to achieving these results.

If the hypothesis tested by the model that the low frequency deflections in the neurophysiological data actually represent important events in the processing of optic flow and attention is correct, then the number and latency of subpeaks, should remain the same for each experimental condition. The fact that each data curve (Figure 1) is the result of averaging a large population of neurons over many trials suggests this may be the case. However, if the undulation properties vary over more trials, as would be the case if the undulations are just noise, our hypothesis would be incorrect. In our “Windowed” simulation, we discovered that increasing the window size had the effect of compressing the temporal spacing of and at times merging the average population peaks together. We therefore predict that characteristics of the low frequency deflections, such as number of subpeaks, change when the neuron population size over which one averages changes, but the deflection properties should remain fixed for a constant sample size.

Although our model successfully reproduces many results of Dubin and Duffy (2007), the model cannot simulate one of the employed paradigms. In Experiment 2 of Dubin and Duffy (2007), monkeys observed behaviorally relevant and irrelevant trials much like those described the present paper. However, in behaviorally irrelevant trials, monkeys were presented with one of four shapes rather than a strictly spatial prime prior to the onset of the optic flow. The monkeys were trained to saccade to its location when a grid of all four shapes appeared. The shape was flashed at the fixation point prior to optic flow onset and would reliably appear in a fixed location different from the fixation point in the grid of four shapes after the optic flow was presented. Therefore, the monkey could learn a specific shape-to-space mapping. In Experiment 3, Dubin and Duffy randomized the location of the shape in the grid to preclude the monkeys from determining the saccade location prior to the shape grid appearance and thereby preventing a space-to-space mapping from forming. In both cases, the neuronal tuning curves qualitatively match those shown in Figure 6: behaviorally relevant and near trials elicited greater average firing in MSTd neurons than in behaviorally irrelevant and far trials, respectively, when the optic flow FoE was nearby the preferred FoE location. Our model cannot simulate the shape paradigm because it does not implement the learning factors required by the monkeys to perform the task. Due to the qualitative similarity between the neuronal tuning curves in the strictly spatial and shape priming paradigms, however, the underlying attentional signal and MSTd dynamics may be similar. If the model was updated to include a learning mechanism to associate a shape with a particular spatial location, we believe our model would simulate the results from Experiments 2 and 3.

As stated previously, we obtained qualitatively similar results for all types of attention when acting within a realistic parameterization of the physiological timing. However, with multiplicative attention, when we employed a different parameterization and slowed down the growth of each unit's integration of its inputs, the relative timing between peaks increased exponentially. This result is because multiplicative modulation of the bottom–up signal gives rise to exponential amplification over time. If input amplitude were logarithmically transformed, the MSTd population peak timings would again be linear. A logarithmic scaling of MSTd inputs is consistent with cortical magnification factor (Elder et al., 2009). If attention acts multiplicatively in cortex and the cortical magnification factor logarithmically transforms the sensory signal, then our analysis would be compatible.

Our results suggest that when attention is engaged during visually-guided navigation, recurrent competition in primate area MSTd modulates the time the population will take to reach its highest activity, and individual neurons tuned to the optic flow FoE, when attended, exhibit higher firing rates. The model predicts that saccade planning and attention modulate the temporal behavior of MSTd neurons, which may affect decision making when primates navigate in the environment. More work needs to be done to understand how primates engage attention in more ecologically relevant scenes, and with independently moving objects. Evidence exists that heading is useful for steering and as such, our model predicts that under divided attention the competition in MSTd will take longer and the time required to make confident steering decisions will also be longer. Recent neurophysiological evidence exists that steering according to an independently moving object or by surrounding optic flow alters the responses of MSTd neurons (Kishore et al., 2011). These findings are consistent with the model results showing that attention to different aspects of the environment changes the response properties of MSTd neurons.

### Conflict of interest statement

The authors declare that the research was conducted in the absence of any commercial or financial relationships that could be construed as a potential conflict of interest.
